# Prevalence and Possible Predictors of Drug-Resistant Pseudomonas aeruginosa in External Ocular Infections: A Single-Center, Retrospective, Cross-Sectional Study

**DOI:** 10.7759/cureus.8082

**Published:** 2020-05-13

**Authors:** Ken-ichi Sato

**Affiliations:** 1 Ophthalmology, Nikko Memorial Hospital, Muroran, JPN

**Keywords:** pseudomonas aeruginosa, anti-infective agents, risk factors, eye infections, drug resistance

## Abstract

Objective

Ocular infections due to multi-drug-resistant Pseudomonas aeruginosa (MDRP) have been reported in recent years. This study was undertaken to determine the culture-positivity rate of P. aeruginosa and drug resistance among patients with suspected external ocular infections and to predict systemic risk factors for drug resistance in P. aeruginosa.

Methods

This retrospective, single-center, cross-sectional study involved 781 consecutive patients who provided samples for aerobic culture to test for a suspected external ocular infection. DRP was defined as a strain resistant to one or two of three antibiotics, levofloxacin, gentamicin, and imipenem; MDRP was defined as that which was resistant to all three.

Results

Among 108 patients in whom gram-negative bacilli were observed, P. aeruginosa was isolated from nine patients, including three DRP-positive cases; no MDRP was isolated. P. aeruginosa was not isolated from those <69 years of age. Among patients in whom gram-negative bacilli were detected, the isolation rate of P. aeruginosa was 0 for patients aged ≤64 and 0.1 for those aged ≥65, indicating a significant difference. For patients with gram-negative bacilli, the DRP-positivity rate was significantly higher for hospitalized patients than outpatients. Thus, in addition to being geriatric, being hospitalized was a risk factor for DRP infection among patients with gram-negative bacilli. All the P. aeruginosa strains isolated were susceptible to colistin.

Conclusions

Our findings may suggest that once gram-negative bacilli are isolated in an elderly hospitalized patient, possible DRP infection and the topical use of colistin should be taken into consideration even before the results of culture and susceptibility testing are obtained.

## Introduction

*Pseudomonas aeruginosa* is a common gram-negative bacillus often found in moist environments. *P. aeruginosa* rarely causes infectious disease in healthy individuals but is responsible for about 10%-20% of nosocomial infections such as sepsis in intensive care units and wound infections, as well in burns and cystic fibrosis [1,2]. Generally, *P. aeruginosa* is resistant to many antibiotics but is known to be susceptible to three classes of antimicrobials: fluoroquinolones, carbapenems, and aminoglycosides.

Since the late 1980s, more strains resistant to three or more classes of antimicrobials have emerged (specifically, fluoroquinolones, carbapenems, and aminoglycosides), and have been termed multiresistant or multi-drug-resistant *P. aeruginosa* (MDRP) [3]. The incidence of MDRP infections is still increasing and is associated with increased morbidity, mortality, and costs [4,5].

In terms of ocular infections, *P. aeruginosa* can cause conditions such as keratitis and endophthalmitis [6,7]. Although ocular MDRP infections have been reported in recent years, only a few studies have investigated focal risk factors for these ocular infections [8-10].

In light of these issues regarding MDRP, the susceptibility of all isolated *P. aeruginosa* strains to colistin, which has been reported to be effective for treating MDRP infections, was investigated in the ophthalmology department of Nikko Memorial Hospital between April 2013 and September 2018 [11]. Subsequently, this retrospective study was undertaken with the aim of determining the culture-positivity rate of *P. aeruginosa *and drug resistance among patients with suspected external ocular infections and to clarify systemic risk factors for drug resistance in *P. aeruginosa*.

## Materials and methods

This retrospective, single-center, cross-sectional study involved 781 consecutive patients who visited the Department of Ophthalmology, Nikko Memorial Hospital (Muroran, Hokkaido, Japan) between April 2013 and September 2018, and provided samples (conjunctival swabs or pus out of the lacrimal sac) for aerobic culture to test for a suspected external ocular infection (Table 1).

**Table 1:** Characteristics of 781 patients with external ocular infection All inpatients were hospitalized for conditions other than ophthalmic disorders.

Sex	Age (years)	Outpatient/inpatient (n)
290 males/491 females	64 ± 26 (mean ± SD); range 0-97; median 72	712/69

Informed consent was obtained from all patients before the examination. The study adhered to the tenets of the Declaration of Helsinki and was approved by the Ethics Committee of Nikko Memorial Hospital.

To identify clinical risk factors, a systematic review of patients' medical history was carried out by interview in addition to obtaining information from medical records of the hospital. Bacterial culture, identification, and susceptibility testing were outsourced to Daiichi Kishimoto Clinical Laboratories, Inc. (Sapporo, Hokkaido, Japan), where the M100-S19 guidelines from the Clinical and Laboratory Standards Institute (Wayne, PA) were used for interpretation of the antimicrobial susceptibility results. For 142 patients with two or more culture tests, only the first result was included in this study; 220 tests were ultimately excluded.

In this study, DRP was defined as a strain resistant to one or two of three antibiotics, levofloxacin, gentamicin, and imipenem; MDRP was defined as that resistant to all three.

Data analysis was performed using StatView 5.0 (SAS Institute, Inc., Cary, NC) with significance set at 5%. Fisher’s exact test was used for statistical analysis.

## Results

**Figure 1:**
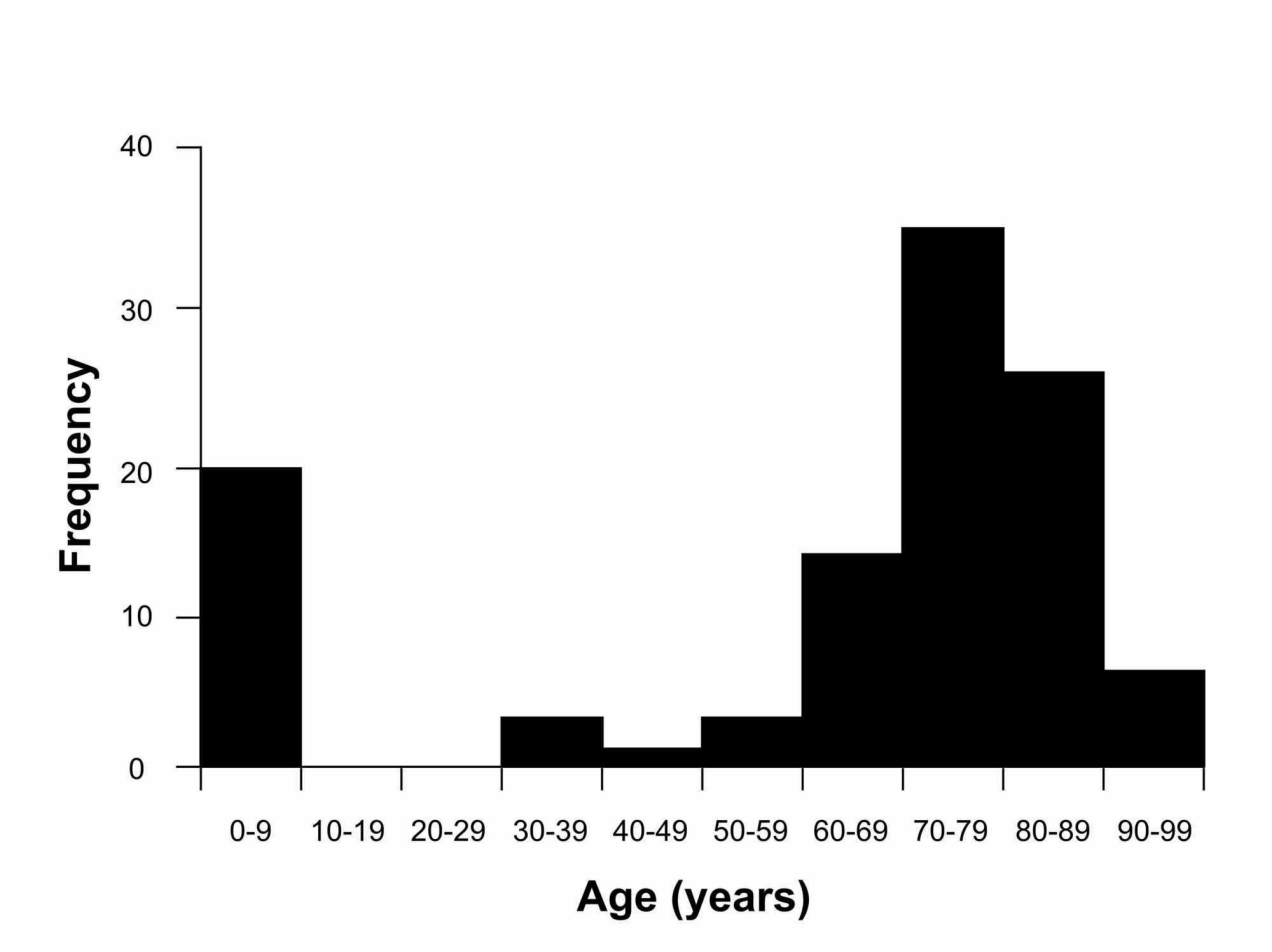
Age distribution of patients in whom gram-negative bacilli were isolated

Gram-negative bacilli were observed in 108 patients, among whom 12 were hospitalized for conditions other than ophthalmic disorders. Those identified as being infected with gram-negative bacilli were divided into two groups by age: the first ≤6 years of age and the second aged ≥32 years (Figure 1).

*P. aeruginosa* was isolated from nine patients, all of whom were ≥69 years of age (Table 2). When gram-negative bacilli were detected, the isolation rate of *P. aeruginosa* was 0 (0/34; 95% CI 0-0.08) for patients aged ≤64 and 0.1 (9/74; 95% CI 0.06-0.2) for those aged ≥65, indicating a significant difference (*P* = 0.028).

**Table 2:** Patients in whom Pseudomonas aeruginosa was isolated LVFX, levofloxacin; GM, gentamicin; IMP, imipenem; CL, colistin; R, resistant; S, susceptible; I, intermediate.

Patient no.	Age (years)/sex	Ophthalmic disorder	Background	Inpatient	Susceptibility			
					LVFX	GM	IPM	CL
1	72/female	Dacryocystitis	Granulomatosis with polyangiitis, immunosuppressants	No	R	S	S	S
2	69/male	Dacryocystitis	Rhinosinusitis	No	S	S	S	S
3	80/female	Bacterial keratitis	Peripheral ulcerative keratitis, topical steroid	No	S	S	S	S
4	81/ male	Exposure keratitis	Cerebral infarction, dysphagia, gastrogavage	Yes	R	I	S	S
5	74/female	Conjunctivitis	Esophageal and pharyngeal cancer, chemotherapy	No	S	S	S	S
6	95/female	Conjunctivitis	Breast cancer, gastrointestinal stromal tumor, cerebral infarction, gastrogavage	Yes	S	S	S	S
7	76/male	Conjunctivitis	Abdominal aortic aneurysm, fungemia	Yes	R	S	R	S
8	84/female	Lachrymal canaliculus obstruction	Lacrimal hyposecretion	No	S	S	S	S
9	78/male	Nasolacrimal duct obstruction	Rhinosinusitis, diabetes mellitus	No	S	S	S	S

DRP was isolated from three patients: one outpatient and two inpatients. No MDRP was isolated in this study. Analyzing the patients with gram-negative bacilli, the DRP-positivity rate was 0.2 (2/12; 95% CI 0.02-0.5) for hospitalized patients and 0.01 (1/96; 95% CI 0.0003-0.06) for outpatients, and this difference was significant (*P* = 0.032).

All the *P. aeruginosa* strains were susceptible to colistin.

## Discussion

Because bacterial properties can change from year to year, guidelines sometimes have to be developed in a relatively short period of time using only a small number of cases. Especially in single-institution studies, it may take time to collect enough positive cases. The current small study is considered to have significance as a method for developing a countermeasure for clinically important bacteria at each facility.

Several risk factors for nosocomial systemic infections caused by MDRP have been reported including age; severity index; being bedridden; transfer from other units; nasogastric feeding; urinary catheterization; mechanical ventilation; intensive care unit stay; and exposure to broad-spectrum antibiotics such as fluoroquinolones, aminoglycosides, and beta-lactams including carbapenems [12-14]. However, it is often difficult to readily obtain this systematic information because patients with suspected MDRP infection tend to have a long medical history and would have received various treatments in multiple hospitals and departments. This impedes immediate clinical decision-making in outpatient care. However, the factor of being hospitalized, which was investigated in the present study, can be easily determined without examining a complicated medical history.

In this study, among patients in whom gram-negative bacilli were isolated, the prevalence of DRP was higher in those who were hospitalized and elderly. The factor of being hospitalized may represent various other known risk factors associated with intensive medical interventions. These two factors, being hospitalized and being elderly, could possibly be adopted as simple predictors of DRP.

Colistin was used in the 1960s and 1970s for treating infections caused by gram-negative bacteria. In spite of the high susceptibility of these bacteria to colistin, it has not been used widely since the early 1980s mainly because of its nephrotoxicity and the subsequent emergence of other broad-spectrum antimicrobials [15]. Later, in addition to its use against gram-negative rods including MDRP in systemic disorders, colistin was refocused as an antimicrobial against MDRP in ocular infections [8,10,11,16].

All the DRP strains isolated in the present study were susceptible to colistin. Thus, our findings may suggest that once gram-negative bacilli are isolated in an elderly hospitalized patient, a possible DRP infection and the topical use of colistin should be taken into consideration even before the results of culture and susceptibility testing are obtained.

Although colistin is usable particularly in countries and regions where it is commercialized as an ophthalmic formulation, careful and reasonable use of colistin is necessary to minimize the induction of colistin-resistant *P. aeruginosa*. Understandably, to prevent accelerating acquisition of resistance, caution should also be exercised in the ongoing empirical use of other classes of antimicrobial agents such like fluoroquinolones and aminoglycosides.

This study has certain limitations. First is that because of the single-center, cross-sectional design, the study did not investigate regional variations in bacterial infections or epidemiological changes over time. Therefore, the results are not universally applicable. Second, the number of patients with *P. aeruginosa* infection was small, and so a larger multi-center study is required.

## Conclusions

In summary, 781 consecutive patients provided samples for aerobic culture to test for a suspected external ocular infection. Among 108 patients in whom gram-negative bacilli were observed, *P. aeruginosa* was isolated from nine patients, including three DRP-positive cases; no MDRP was isolated. In addition to being geriatric, being hospitalized was a risk factor for DRP infection among patients with gram-negative bacilli. All the *P. aeruginosa* strains isolated were susceptible to colistin.
